# Primary mesenchymal stem cells in human transplanted lungs are CD90/CD105 perivascularly located tissue-resident cells

**DOI:** 10.1136/bmjresp-2014-000027

**Published:** 2014-05-17

**Authors:** Sara Rolandsson, Annika Andersson Sjöland, Jan C Brune, Hongzhe Li, Moustapha Kassem, Fredrik Mertens, Albert Westergren, Leif Eriksson, Lennart Hansson, Ingrid Skog, Leif Bjermer, Stefan Scheding, Gunilla Westergren-Thorsson

**Affiliations:** 1 Department of Experimental Medical Science, Lung Biology Unit, Lund University, Lund, Sweden; 2 Lund Stem Cell Center, Lund University, Lund, Sweden; 3 Department of Endocrinology, Molecular Endocrinology Laboratory (KMEB), University of Southern Denmark, Odense, Denmark; 4 Department of Clinical Genetics, University and Regional Laboratories, Lund University, Lund, Sweden; 5 School of Health and Society, Kristianstad University, Lund, Sweden; 6 Department of Respiratory Medicine and Allergology, Lund University and Skåne University Hospital, Lund, Sweden; 7 Department of Hematology, Skåne University Hospital, Lund, Sweden

**Keywords:** Lung Transplantation

## Abstract

**Background:**

Mesenchymal stem cells (MSC) have not only been implicated in the development of lung diseases, but they have also been proposed as a future cell-based therapy for lung diseases. However, the cellular identity of the primary MSC in human lung tissues has not yet been reported. This study therefore aimed to identify and characterise the ‘bona fide’ MSC in human lungs and to investigate if the MSC numbers correlate with the development of bronchiolitis obliterans syndrome in lung-transplanted patients.

**Methods:**

Primary lung MSC were directly isolated or culture-derived from central and peripheral transbronchial biopsies of lung-transplanted patients and evaluated using a comprehensive panel of in vitro and in vivo assays.

**Results:**

Primary MSC were enriched in the CD90/CD105 mononuclear cell fraction with mesenchymal progenitor frequencies of up to four colony-forming units, fibroblast/100 cells. In situ staining of lung tissues revealed that CD90/CD105 MSCs were located perivascularly. MSC were tissue-resident and exclusively donor lung-derived even in biopsies obtained from patients as long as 16 years after transplantation. Culture-derived mesenchymal stromal cells showed typical in vitro MSC properties; however, xenotransplantation into non-obese diabetic/severe combined immunodeficient (NOD/SCID) mice showed that lung MSC readily differentiated into adipocytes and stromal tissues, but lacked significant in vivo bone formation.

**Conclusions:**

These data clearly demonstrate that primary MSC in human lung tissues are not only tissue resident but also tissue-specific. The identification and phenotypic characterisation of primary lung MSC is an important first step in identifying the role of MSC in normal lung physiology and pulmonary diseases.

**Key messages**

- Can primary mesenchymal stem cells be identified and isolated from human lung tissue and where are they localised?
- Primary mesenchymal stem cells in human lungs are CD105+/CD90+ cell that are perivascularly located tissue-resident cells.
- The identification and phenotypic characterisation of primary lung MSC is an important first step in identifying the role of MSC in normal lung physiology and pulmonary diseases.

## Introduction

Mesenchymal stem cells (MSC) are multipotent cells, which have been isolated from a variety of tissues such as bone marrow, cord blood, fat and lungs.^1–4^ In vitro, clonogenic cells, which are denoted as colony-forming units, fibroblast (CFU-F),^5^ can be assayed as plastic adherent cells giving rise to fibroblastic colonies. These CFU-F cells are considered to reflect the primary MSC and, on further proliferation in culture, their descendants make up the extensively studied cultured mesenchymal stromal cells.^6^ ^7^

MSC have a number of intriguing properties, that is, high proliferation potential, differentiation capacity into different mesenchymal cell types such as adipocytes, osteoblasts and chondrocytes, capacities to produce a variety of different cytokines, chemokines, extracellular matrix and microvesicles, as well as potent immunomodulatory function.^5^ ^8–11^

Owing to these features, MSC have become promising candidates for cellular therapies. For example, with regard to pulmonary diseases, MSC have been demonstrated to reduce inflammation and fibrosis in lung injury mouse models.^12^ ^13^ MSC have also been proposed as potential precursors for fibroblasts/myofibroblasts, thereby contributing to the development of fibrotic obliteration of small airways.^14^ ^15^

Fibrotic obliteration of the peripheral airways (obliterative bronchiolitis) is the hallmark of chronic rejection after lung transplantation, affecting approximately 50–60% of patients within 5 years after transplantation and constituting the main cause of long-term morbidity and mortality. Since obliterative bronchiolitis is a patchy disease, the term bronchiolitis obliterans syndrome (BOS) is used clinically and is defined by a decline in lung function and is measured by spirometry and further graded from one to three based on the lung function results.^16^ ^17^

Previous studies have demonstrated that donor-derived MSC are present in the bronchoalveolar lavage (BAL) fluid from lung-transplanted patients.^18^ Furthermore, MSC were successfully culture isolated from human lung tissue.^2^ ^19^ However, little is known about cellular identity and properties of primary ‘bona fide’ MSC in the lung, which is the key information required to investigate the function and physiological/pathophysiological role of MSC in situ.

We demonstrate—for the first time—the identity, phenotype and localisation of primary MSC in lung tissues of lung-transplanted patients from central and peripheral transbronchial biopsies. Our results show that lung MSC are tissue-resident cells with typical MSC properties except for in vivo bone formation, thus indicating that pulmonary MSC represent a population of tissue-specific stromal progenitor cells. Furthermore, our results indicate that lung-derived MSC—in contrast to what has been reported for MSC from BAL—do not correlate with the development of BOS in lung-transplanted patients.

## Methods

### Isolation of lung cells from lung biopsies

Central (bronchial wall) and peripheral transbronchial (parenchymal tissue) biopsies were collected at different time points after transplantation (range 3 months–16 years), and biopsies were preferentially taken from the right lower lobe. Lung function was measured by standard spirometry and BOS grades were defined according to the International Society for Heart and Lung Transplantation guidelines (see online supplementary table S1).^20^ All patients gave their written informed consent to participate in the study (2005-560). Fresh lung biopsies were cut into smaller pieces and then further dissociated by enzymatic digestion with collagenase type I, 300 U/mL (Gibco BRL, Paisley, USA), hyaluronidase, 1 mg/mL (Fisher Scientific) and DNAse (Qiagen, Solna, Sweden) in Dulbecco’s phosphate buffered saline (DPBS). After washing, lung cells were seeded in NH expansion medium (Miltenyi Biotec, Bergisch Gladbach, Germany) supplemented with 1% antibiotic antimycotic solution (Sigma Aldrich, Stockholm, Sweden) at 37°C, 5% CO_2_ for generation of lung-derived MSC. Medium was changed after 3 days and weekly thereafter. MSC were passaged with 0.05% trypsin-EDTA (Invitrogen, Lidingö, Sweden) at 70–90% confluence.

### CFU-F assay

The frequency of CFU-F in lung cells was determined as described previously.^21^ ^22^ Briefly, lung cells were isolated as above and plated in a median of 4 (range 1.0–6.0) wells of a six-well plate at 3×10^4^ and 4×10^4^ cells/well. Complete medium changes were performed after 3 and 7 days. On day 14, cells were washed with DPBS, fixed with methanol and stained with 0.5% or 0.1% crystal violet. CFU-F were enumerated microscopically (Nikon TMS microscope (Nikon, Tokyo, Japan) equipped with Infinity1 camera (BergmanLabora AB, Danderyd, Sweden)) as colonies with ≥40 fibroblast-like cells. Wells containing more than 50 colonies were counted as more than 150 CFU-F/100 000 cells.

### MSC in vitro differentiation assay

Cultured lung-derived MSC were differentiated towards adipocytes, osteoblasts and chondrocytes as described.^21^ ^22^ Briefly, for adipocyte differentiation, cells were cultured under standard conditions until confluency. Thereafter, cells were cultured for another 21 days in AdipoDiff medium (Miltenyi Biotec), and stained with Oil-Red-O following fixation (Sigma Aldrich). For osteoblast differentiation, MSC were cultured in Dulbecco's modified Eagle's medium (DMEM) high glucose/l-glutamine (PAA) containing β-glycerophosphate (Sigma Aldrich), l-ascorbic acid-2-phosphate (Wako Chemicals, Neuss, Germany) and dexamethasone (Sigma Aldrich) for 21 days and calcium deposition was visualised by alizarin red staining (Sigma Aldrich). For chondrocyte differentiation, cell pellets were cultured in complete chondrocyte induction medium consisting of l-ascorbic acid-2-phosphate, pyruvic acid sodium salt (Sigma Aldrich), l-proline (Sigma Aldrich), ITS^+^ culture supplement (BD Bioscience, Erembodegem, Belgium) and TGF-β3 (R&D Systems, Abingdon, UK). After 28 days, pellets were fixed and embedded in OCT compound (Tissue-Tek, Sakura, Zoeterwoude, The Netherlands). The pellets were sectioned and stored at −80°C. For analysis, sections were stained with goat antihuman aggrecan (R&D Systems) and a corresponding secondary antibody (Donkey anti-goat IgG, Texas Red (JackssonEurope, Suffolk, UK)). Nuclei were stained with 4′,6-diamidino-2-phenylindole (DAPI). Pictures of adipocyte and osteoblast differentiation were taken with a Nikon Eclipse TE2000-E microscope equipped with a Nikon DS-U2/L2 USB camera and the chondrocyte differentiation was documented with an Axiovert 200M fluorescence microscope and an AxioCam HRm camera.

### Gene expression analysis of cultured lung MSC

Cultured lung MSC (passage 4) were harvested with 0.05% trypsin-EDTA and total RNA was isolated with the RNeasy mini kit (Qiagen, GmBH, Hilden, Germany). The amount of RNA was measured using NanoDrop ND-100 (Nano Drop Technologies, Delaware, Maryland, USA) and 1 μg RNA was used for cDNA synthesis. Superscript II (Invitrogen, Carlsbad, California, USA) was used to reverse-transcribe RNA and the cDNA was stored at −80°C. Then the cDNA was mixed with primers (see online supplementary material) and Fast CYBR Green Master Mix 2X (Applied Biosystems). Reverse transcription-PCR reactions were performed with the StepOnePlus Real Time PCR system (Applied Biosystems) starting at 50°C for 2 min and 95°C for 2 min, followed by 45 cycles of 95°C for 15 s, 60°C for 25 s and 73°C for 30 s, and finished by 95°C for 15 s, 70°C for 15 s and 98°C for 15 s. Relative quantifications were performed according to 2^−Δ ΔCt^ method using GAPDH as a housekeeping gene and centrally derived MSCs as reference gene.

### MSC in vivo differentiation assay

In vivo transplantation of lung-derived MSC was performed as described previously.^23^ Briefly, central and peripheral lung MSC from two patients were harvested, incubated with hydroxyapatite/tricalcium phosphate (HA/TCP) ceramic powder overnight at 37°C in 5% CO_2_ and 500 000 cells were implanted subcutaneously into 8-week-old female non-obese diabetic/severe combined immunodeficient (NOD/SCID) mice (four implants per culture). Implants were removed after 8 weeks, fixed, decalcified and paraffin embedded. Implants from one animal had to be removed 4 days earlier due to development of thymic lymphoma. Sections were stained with H&E and analysed as described.^24^ Photomicrographs were taken with a Leica DM4500B microscope equipped with a motorised stage from Märzhäuser Wetzlar GmbH and a Leica DFC300 camera, and controlled by the Surveyor software (Objective Imaging).

### Immunophenotyping

Cells were detached with 0.05% trypsin-EDTA and blocked with phosphate buffered saline (PBS) containing 1% fetal bovine serum (FCS; Gibco BRL), 3.3 mg/mL human immunoglobulin (Gammanorm; Octapharma, Stockholm, Sweden) and 0.1% sodium azide. Cells were labelled with different combinations of the following direct conjugated antibodies: anti-CD105-FITC, anti-CD34-FITC, anti-HLA-DR-FITC, anti-CD31-FITC, anti-CD73-PE, anti-CD14-PE, anti-19-PE, anti-HLAclass I-PE, anti-CD146-PE, anti-CD90-APC, anti-CD45-APC (all from BD Bioscience) and anti-CD271-APC (Miltenyi Biotec). Corresponding isotype controls were all from Becton Dickinson. Before analysis, cells were stained with 7-amino-actinomycin D (1 μL/mL; Sigma Aldrich) for dead cell exclusion. Samples were analysed on an FACSCalibur (BD Bioscience). Data acquisition and analysis were performed using CellQuest (BD Bioscience) and FlowJo software (Tree star, Ashland, Oregon, USA).

### Fluorescence-activated cell sorting of primary lung cells

Following blocking with PBS containing 1% FCS and 3.3 mg/mL human immunoglobulin, single lung cell suspensions were stained with combinations of the following antibodies: anti-CD90-APC/FITC and anti-CD105-FITC/APC (BD Bioscience) or anti-CD146-PE with anti-CD271-APC. After washing, cells were incubated with 7-amino-actinomycin D (1 μL/mL) and sorted on an FACSAria I or FACSAria III cell sorter (both from BD Bioscience). Single stained cells were used to set up sorting gates and doublets were excluded by gating on SSC-H versus SSC-W and FSH-H versus FSC-W. Sorted cells were collected in NH medium supplemented with 1% antibiotic antimycotic solution and gentamicin (Gibco BRL) and assayed for CFU-F as described above; colonies with ≥20 cells were counted as CFU-F. In total, 11 sorting experiments were performed on CD105 and CD90 stained cells. However, five of the experiments were not evaluable because of insufficient cell yield after sorting.

### Karyotyping and X and Y chromosomes fluorescence in situ hybridisation of lung-derived MSC

Central and peripheral MSC from seven sex-mismatched lung-transplanted patients were harvested, fixed in methanol:glacial acetic acid (3:1) and spread on slides. After spreading, the slides were kept at 60°C overnight and then treated with 2T (2×saline–sodium citrate (SCC) buffer with 0.05% Tween-20 (Sigma Aldrich)) at 60°C. Slides were then incubated with 20 mg/mL pepsin in 0.01M HCl at 37°C for 10 min, washed and incubated with 1% formaldehyde in PBS, washed and dehydrated. LSI SRY and CEP X probes (Vysis, Downers Grove, Illinois, USA) were added to the slides and denaturation was performed at 74°C. Hybridisation was carried out overnight at 37°C in a moist chamber. Posthybridisation washes were carried out in 0.4T (0.4×SSC with 0.05% Tween-20) in a 72°C water bath. Slides were dehydrated and mounted with DABCO/DAPI (DABCO, Sigma Aldrich; DAPI, ROCHE, Bromma, Sweden). One hundred nuclei were analysed per sample.

Karyotyping of the MSC cultures was performed as described.^25^ Briefly, cultured MSC derived from central biopsies of two sex-mismatched lung-transplanted patients were arrested in metaphase with 0.04 µg/mL colcemid. Then the cells were fixed, prepared on slides and chromosomes were G-banded using Wright’s stain. Twenty-five metaphases were analysed per sample. Images were taken with a Zeiss Axioplan 2 microscope (Carl Zeiss AG, Oberkochen, Germany) using CytoVision software (Leica Biosystems, Nussloch, Germany).

### Immunohistochemistry

Paraffin-embedded tissue samples were sectioned into 4.5 μm thick sections. Sections were rehydrated and antigen retrieval was performed in high pH buffer (EnVision FLEX target retrieval solution (K8004, Dako, Glostrup, Denmark)) in microwave. Endogenous peroxidase activity was quenched with 0.5% H_2_O_2_. CD105 was detected by an anti-CD105 mouse monoclonal antibody (Clone 4G11, Leica Microsystems, Newcastle, UK) diluted in an EnVisionFLEX antibody diluent (K8006, Dako) and the signal was enhanced with the Tyramid Signal Amplification kit (T20912, Molecular Probes) and visualised with EnVision labelled ploymere HRP (K4001, Dako). CD90 expression was detected with an anti-CD90 rabbit monoclonal antibody (Clone EP56, Epitomics, California, USA) diluted in an EnVisionFLEX antibody diluent and visualised with a goat antirabbit Alexa Fluor 555-conjugated secondary antibody. Nuclei were stained with DAPI and sections were mounted. Staining was absent in sections using isotype control antibodies (see online supplementary figure S5A–F). Pictures were taken with a Nikon TE200E microscope equipped with a high-resolution camera Nikon DXM 1200C using the NIS-Elements AR V.3.0 system (Nikon).

### Statistics

CFU-F data were analysed statistically by using the Mann-Whitney U test to compare central and peripheral CFU-F numbers. Results are presented as median and range. Analyses were performed with the GraphPad Prism software V.5.0c. Two Kaplan-Meier analyses with BOS as the outcome variable and time, status and groups CFU-F central (<50 = 0 vs >50=1) and CFU-F peripheral (<10=0 vs >10=1), respectively, were conducted.^26^ Furthermore, data were analysed for correlation by a predictive model built on multivariate logistic regression. Kaplan-Meier and regression analyses and correlation analysis were conducted with the IBM SPSS Statistics V.19.0 software package. p-Values ≤0.05 were considered as significant. A detailed description of the statistical analysis is provided in the online supplementary material.

## Results

### Primary MSC can be isolated from central and peripheral transbronchial biopsies in lung-transplanted patients

**Figure 1:**
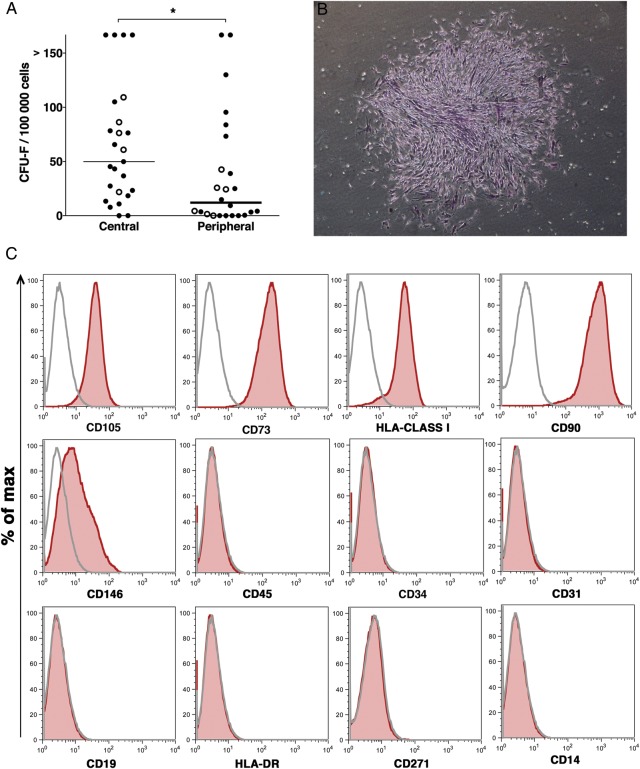
Lung-derived cultured mesenchymal stem cells (MSC) show a typical surface marker profile and have a higher colony-forming unit, fibroblast (CFU-F) frequency in lung MSC derived from central biopsies compared with peripheral transbronchial biopsies. Primary cells were isolated from the central and peripheral transbronchial lung biopsies of 27 lung-transplanted patients. Single-cell suspensions were assayed for CFU-F content under standard conditions for 14 days. (A) Primary lung cells derived from central biopsies displayed a significantly higher CFU-F frequency compared with peripheral transbronchially derived cells (p=0.0254). Median CFU-F frequencies of individual biopsies are shown as colonies/100 000 seeded cells. Closed circles represent CFU-F numbers from biopsies of individual patients undergoing lung biopsies once after transplantation, whereas open circles represent CFU-F numbers of repetitive biopsies of patients undergoing bronchoscopies at different time points after transplantation. The Mann-Whitney U test was used to calculate p values. *p<0.05. Colonies with ≥40 cells were counted as CFU-F. (B) A typical lung-derived colony stained with crystal violet. (C) Cultured lung MSC (passages 3 and 4) were harvested, stained with surface marker antibodies and analysed with flow cytometry. A representative surface marker profile from central lung-derived MSC of one patient is presented. Sample: red shaded area. Isotype control: grey line.

Primary mesenchymal stem/progenitor cells are considered to be reflected by in vitro clonogenic cells—denoted as CFU-F. In order to determine the mesenchymal progenitor frequency in lung biopsies, single-cell suspensions from central and peripheral transbronchial biopsies of 27 lung-transplanted patients (in some of the patients, biopsies were collected at more than one time point after transplantation) were assayed in standard CFU-F assays (figure 1). Colony formation was observed in all but two central biopsies (n=30 cultures) and in 24 of 31 cultures from peripheral transbronchial biopsies. As shown in figure 1A, mesenchymal progenitor frequency was significantly higher (p=0.0254) in central biopsies (median 49.86 CFU-F/10^5^ seeded cells; range 0.0–166.7) compared with peripheral tissues (median 12.05; range 0.0–166.7). The median intra-assay variations of centrally derived cells were 9.8% (range 0–173.2) and 8.6% (range 0–173.2) at a seeding density of 3×10^4^ and 4×10^4^ cells/well, respectively. For peripherally derived cells, the median intra-assay variations were 9.7 (range 0–173.2) and 5.1 (range 0–173.2) at a seeding density of 3×10^4^ and 4×10^4^ cells/well, respectively. The mesenchymal colonies assayed from lung tissues showed typical CFU-F morphology as shown in figure 1B.

The Kaplan-Meier analysis revealed no significant differences between the number of central CFU-F in relation to time to event (BOS; mean 2.79 years, 95% CI 1.52 to 4.05 and mean 3.01 years, 95% CI 0.99 to 5.03, respectively, p=0.508; see online supplementary figure S1A). In the second Kaplan-Meier analysis, no differences between the number of peripheral CFU-F in relation to time to event (BOS) were seen (mean 2.97 years, 95% CI 1.69 to 4.26 and mean 3.25 years, 95% CI 1.33 to 5.17, respectively, p=0.541; see online supplementary figure S1B). Furthermore, a logistic regression (enter model) analysis demonstrated that none of the tested independent variables were significantly correlated with the number of CFU-F in central or peripheral biopsies (see online supplementary table S2).

### Lung-derived mesenchymal stromal cells show a typical MSC surface marker profile and multilineage differentiation capacity in vitro but lack bone differentiation capacity in vivo

On further proliferation in culture, CFU-F give rise to the so-called cultured mesenchymal stromal cells.^1^ ^18^ ^27^ Immunophenotyping of lung-derived cultured mesenchymal stromal cells (passages 3 and 4) using multicolour flow cytometry showed a typical MSC surface marker profile, that is, lung-derived MSC were positive for CD105, CD73, CD90, HLA-class I and CD146, but lacked expression of CD45, CD34, CD14, CD19, HLA-DR, CD31 and CD271 (figure 1C). There were no differences in surface marker expression between central and peripheral transbronchial biopsy MSC (see online supplementary figure S2).

**Figure 2:**
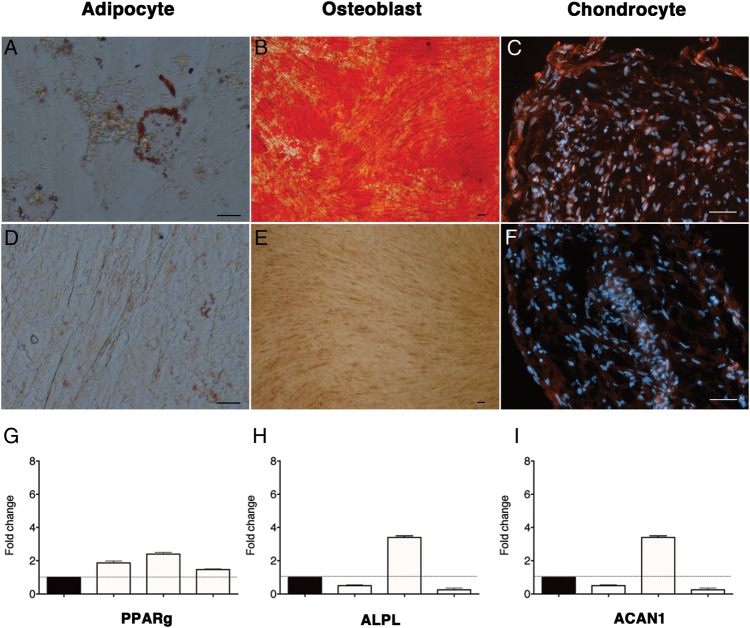
Lung-derived mesenchymal stem cells (MSC) display in vitro multilineage potential. MSC derived from central and peripheral transbronchial lung biopsies obtained from six lung-transplanted patients were seeded in an appropriate differentiation-induction medium. Lung-derived MSC differentiated into adipocytes (A), osteoblasts (B) and chondrocytes (C). Adipocytes were stained with Oil-Red-O staining (A and D), osteoblasts with alizarin red (B and E) and chondrocytes with an antiaggrecan antibody (C and F). Control cells were cultured in normal growth medium (D–F). Scale bare for adipocytes, osteoblast and chondrocyte pictures represents 20, 100 and 50 μm, respectively. To confirm in vitro differentiation, we performed reverse transcription-PCR on cultured MSC obtained from three lung-transplanted patients (G–I). Fold change in mRNA expression of peripheral transbronchially derived cells (white) is shown for peroxisome proliferator-activated receptor γ (PPARG; G), alkaline phosphatase (ALPL; H) and aggrecan (ACAN; I) using centrally derived cells (black) as the reference gene.

**Figure 3:**
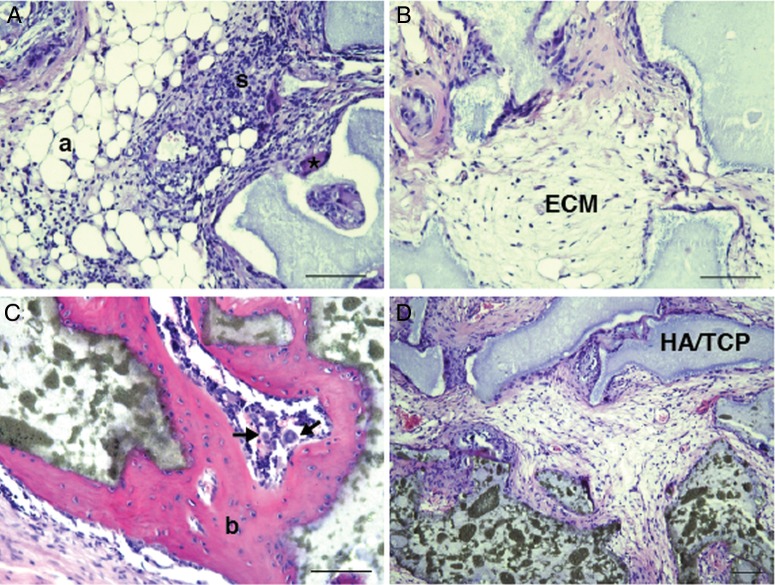
Lung-derived mesenchymal stem cells (MSC) display impaired bone formation capacity in vivo. Cultured MSC derived from two lung-transplanted patients were subcutaneously transplanted (together with hydroxyapatite/tricalcium phosphate (HA/TCP) particles) into NOD/SCID mice. After 8 weeks, the transplants were removed, fixated and stained with H&E (A, B). Lung-derived MSC clearly formed adipocytes (a), stroma with invading haematopoietic cells (s) and an extracellular matrix (ECM). However, bone formation was hardly observable (*) compared with the bone formation (b) seen in the positive control (C, telomerase-immortalised human MSC). Arrow indicates a megakaryocyte. HA implants without cells served as negative controls (D). Scale bars indicate 100 μm.

In vitro differentiation experiments revealed multilineage differentiation potential of lung-derived MSC from central and peripheral transbronchial biopsies, that is, cells differentiated into adipocytes, osteoblasts and chondrocytes. Adipocyte and osteoblast differentiation was observed in four of six and five of six cultures of MSC derived from central and peripheral biopsies, respectively (figure 2A,B). Chondrocyte differentiation was observed for central biopsy-derived cells (in one of three cultures; figure 2C). No differentiation was seen in the negative control (figure 2D–F). In vitro differentiation capacity was further confirmed by gene expression analysis (figure 2G–I). Expression of *PPARG* (peroxisome proliferator-activated receptor γ), ALPL (alkaline phosphatase) and ACAN (aggrecan) was seen in centrally and peripherally derived cultures. The in vivo differentiation potential of lung-derived MSC was investigated by xenotransplantation of cultured MSC together with HA/TCP carrier particles subcutaneously into NOD/SCID mice. Lung-derived MSC formed adipocytes and stromal tissues in vivo. However, bone formation was clearly impaired or even absent in most of the slides evaluated. Generally, only small areas of possible bone tissue were detectable in lung MSC transplanted animals (figure 3A,B). Control telomerase-immortalised bone marrow MSC, on the other hand, showed clear bone formation (figure 3C). HA/TCP carrier particles without cells served as negative controls (figure 3D).

### Lung MSC are tissue-resident

**Figure 4:**
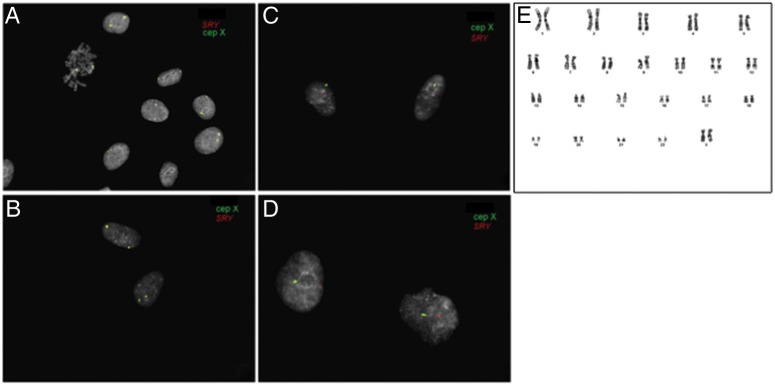
Lung mesenchymal stem cells (MSC) are donor derived and tissue resident. Cultured MSC isolated from central (A and C) and peripheral transbronchial (B and D) biopsies of seven sex-mismatched lung-transplanted patients were harvested and analysed by fluorescence in situ hybridisation. LSI SRY (orange) and CEP X (green) probes were used in order to distinguish donor-derived cells from recipient cells, with the presence of Y and X chromosomes indicated by the red and green signal, respectively. A and B show that the lung-derived MSC from male patient originated from the female lung donor. C and D show male donor MSC in a female recipient. Furthermore, karyotyping was performed on MSC from central biopsies of two patients demonstrating a normal karyotype as exemplified in E.

In order to examine if lung MSC were tissue resident and originated from donor lungs, we performed fluorescence in situ hybridisation (FISH) analysis on cultured central and peripheral transbronchial-derived cells (passages 3 and 4) from sex-mismatched lung-transplanted patients (n=7; figure 4). MSC were isolated from biopsies taken as soon as 3 months, and as late as nearly 16 years after transplantation (see online supplementary table S3). All evaluated MSC samples showed donor sex karyotype (median 97%; range 93–100%; figure 4, see online supplement table S3). There was no difference between MSC derived from central biopsies (figure 4A, C) compared with peripheral transbronchial biopsies (figure 4B, D). In addition, G-band analysis was performed on passage 4 MSC samples, confirming the results of the FISH analysis and furthermore demonstrating that cultured lung-derived MSC had a normal karyotype (figure 4E).

### Primary pulmonary MSC are enriched in the CD90/CD105 cell fraction

**Figure 5:**
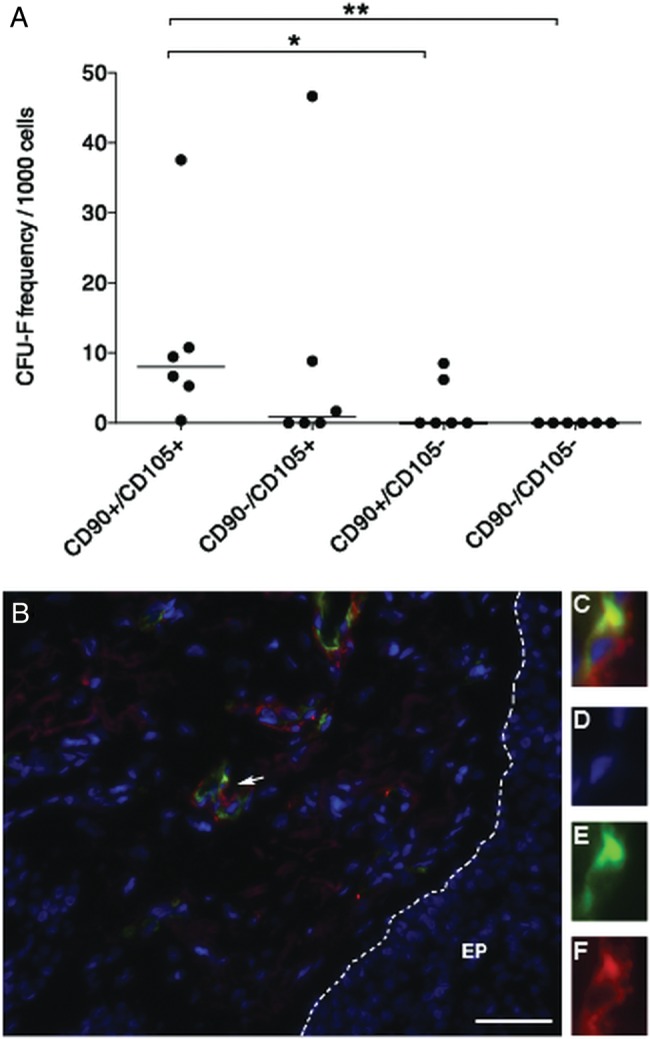
Primary mesenchymal stem cells (MSC) are enriched in the CD90/CD105 double positive cells in human lung tissue and are located perivascularly. (A) Single-cell suspensions were isolated from central and peripheral transbronchial lung biopsies of lung-transplanted patients, stained with direct conjugated antibodies against CD90 (Thy-1) and CD105 (Endoglin), and sorted by fluorescent-activated cell sorting. CD90/CD105, CD90−/CD105, CD90/CD105− and CD90−/CD105− cell populations were sorted and assayed for colony-forming unit, fibroblast (CFU-F) content. Colonies were enumerated after 13 and 14 days in culture, showing that the majority of CFU-F was contained in the CD90/CD105 double positive cell fraction. Colonies with ≥20 cells were counted as CFU-F. Statistical analysis was performed using a non-parametric Mann–Whitney U test. Horizontal lines indicate medians for each sorted cell fraction. *p<0.05, **p<0.01. (B) Paraffin embedded sections from lung-transplanted patients were stained with antibodies against CD90 and CD105. CD90/CD105 double positive cells were observed in perivascular regions. Arrow indicates a CD90/CD105 cell. (C–F) Enlargement of a CD90 (F) and CD105 (E) double positive cell, the nuclei is stained with 4′,6-diamidino-2-phenylindole (D). EP, small airway epithelium. Scale bar represents 20 μm.

Next, we wanted to investigate the phenotype of the primary lung MSC and evaluate if these cells could be isolated directly from lung tissues by fluorescence-activated cell sorting (FACS) based on surface markers previously described for MSC isolation. First, primary lung cells were sorted based on the expression of CD146 and CD271 (n=4), a surface marker combination that has previously been used by us and others for the isolation of primary MSC from human bone marrow.^21^ ^28^ However, and in contrast to bone marrow, lung CFU-F were not only found in the CD271 single positive fractions but also in double negative cells in three of four experiments. In addition, CFU-F were even occasionally found in double positive and CD146 single positive cells, respectively, indicating that CD271/CD146 is not a suitable surface marker combination to identify lung CFU-F (see online supplementary figure S3A–E). On the other hand, when primary lung mononuclear cells were sorted based on CD90/CD105 expression (n=6), CFU-F were consistently found in the double positive fraction (median 8.05 colonies/1000 primary cells, range 0.35–37.59), but only very occasionally in the CD90−/CD105 (median 0.84 colonies/1000 primary cells, range 0.0–46.67) and CD90/CD105− fractions (median 0 colonies/1000 primary cells, range 0.0–8.49). Moreover, CFU-F were not detected in CD90−/CD105− double negative cells (figure 5A).

### In situ localisation of CD90/CD105 cells in human lung tissue

In order to investigate the in situ localisation of resident lung CD90/CD105 CFU-F, sections from lung-transplanted patients were analysed by antibody staining. Generally, only very few CD90/CD105 double positive cells were found, which correlates well with the low number of these cells in the sorting experiments as well as with CFU-F frequencies in lung tissues. In accordance with the reported MSC locations in other organs, double positive CD90/CD105 lung cells were found to be located perivascularly (figure 5B).

## Discussion

MSC have not only been implicated in the development of lung diseases, but they have also been proposed as a future cell-based therapy for lung diseases, such as obliterative bronchiolitis. Despite this potentially important role of MSC in lung physiology, relatively little is known about the cellular identity and biological function of the primary human lung MSC in health and disease. Thus far, only culture-derived stromal cells from BAL fluid,^18^ fetal and adult healthy lung tissues have been investigated.^2^ ^19^ ^29^ We therefore aimed to isolate primary pulmonary MSC in lung tissue from transplanted patients in order to investigate their origin and anatomical localisation within the lung tissue, as well as to characterise their mesenchymal properties in vivo and in vitro.

Our results demonstrate that mesenchymal stem/progenitor cells, as assessed by CFU-F assays, were present in central and peripheral transbronchial biopsies. CFU-F frequencies were significantly higher in the central biopsies indicating, in accordance with what has been reported previously,^30^ that there are differences in lung structure between central and peripheral sections. Interestingly, there was no correlation between CFU-F number and BOS grade, indicating that lung-derived tissue-resident MSC could not be used as a predictor for BOS development. Badri *et al*,^31^ on the other hand, reported previously that the measurement of CFU-F in BAL fluid provided predictive information regarding future BOS onset in lung-transplanted patients. This finding leads to the assumption that CFU-F numbers in transplanted lung tissues might be independent from CFU-F numbers in BAL fluids. However, as we have not directly compared these two cell sources, we cannot draw any definite conclusions at this point of time. Alternatively, lung tissue and BAL MSC might have biological differences and therefore react differently to pathological stress situations. Certainly, this is an important consideration which is worth addressing in future experiments.

One of the fundamental properties of MSC is their multilineage differentiation capacity, which is commonly evaluated only by in vitro assays. Our data show that pulmonary MSC possessed standard multilineage differentiation potential in vitro, thus formally fulfilling the requirements for MSC definition, but they lacked proper bone formation in vivo. This indicates that lung MSC and bone marrow-derived MSC are biologically different as bone marrow MSC robustly formed bone in xenotransplantation experiments.^21^ Based on the fact that the differentiation spectrum of bone marrow-derived MSC also differs from other MSC preparations, such as adipocyte-derived and umbilical vein-derived stromal cells, it is reasonable to conclude that MSC are tissue-specific.^32^

Furthermore, our observation that lung-derived MSC possessed a full three-lineage potential only in vitro, which is most likely due to supraphysiological levels of differentiation-inducing signals in standard in vitro cultures, stresses the importance of in vivo experiments to assess proper differentiation potential.

We analysed lung-derived MSC by XY chromosome analysis and our data clearly demonstrate that pulmonary MSC in lung-transplanted patients were donor derived. Interestingly, different origins of pulmonary MSC have been discussed, with one of the hypotheses being that MSC are recruited from the bone marrow through the circulation.^13^ ^33^ However, our results in accordance with data previously reported by Lama *et al*^18^ provide compelling evidence that lung-derived MSC are tissue-resident cells. Some of the analysed MSC were isolated from biopsies taken as long as nearly 16 years after transplantation, indicating that lung-resident MSC either are very long-lived cells or that they might possess self-renewal capacity, which is certainly an important point given the recent data providing evidence for in vivo self-renewal of murine and human MSC.^28^ ^34^

Until now, only culture-derived lung MSC have been studied and the phenotype of the primary lung MSC has thus far been elusive. We identify—for the first time—the phenotype of primary lung-derived MSC. Using FACS sorting of primary lung cells, we could demonstrate that the CD90/CD105 cell fraction was highly enriched for CFU-F. Based on our previous results on the isolation of CFU-F in primary bone marrow-derived cells, we initially started sorting based on CD271 and CD146 expression,^21^ but this combination did not allow to enrich for lung CFU-F, which furthermore strengthens the tissue specificity of MSC.

Owing to the fact that only limited numbers of cells were available for sorting due to the small size of the tissue biopsies, it was not possible to reanalyse the sorted cell fractions. Therefore, colonies growing from single positive CD105 cells might be the result of sorting impurities. Here, the sorting of a larger number of cells, for example, from lung explants would be necessary, which is certainly an important consideration for future experiments.

On the basis of the identification of the phenotype of primary lung MSC, we then could address the important question of where these cells are located in situ in the lung tissue. Here, our experiments showed that CD90/CD105 double positive cells were found exclusively perivascularly. The perivascular location of the pulmonary MSC is thus in accordance with what has been demonstrated in other organs such as bone marrow.^28^ ^35^ ^36^

Several preclinical studies have evaluated the therapeutic potential of MSC in different lung disease models. Ortiz *et al*^12^, for example, demonstrated that the administration of bone marrow-derived MSC into bleomycin-treated mice resulted in decreased inflammation and collagen deposition. Furthermore, Rojas *et al*^13^ reported an increased production of growth factors, a better survival and reduced inflammation in bleomycin-induced lung fibrosis in mice treated with bone marrow-derived MSC. Bone marrow-derived MSC have already been used clinically in humans for the treatment of inflammatory diseases such as severe graft-versus-host disease in haematopoietic stem cell transplantation.^37^ Autologous bone marrow-derived MSC have also been used to improve graft function in solid organ transplantation and MSC are furthermore under active investigation in non-transplant settings for the treatment of various diseases, such as myocardial infarction^38^ and multiple sclerosis.^39^ Furthermore, bone marrow-derived MSC have been used in clinical trials for treatment of chronic obstructive pulmonary disease where it was proved to be a safe treatment.^40^ Clinical MSC treatment has been reported to be effective and safe and has therefore also been suggested as a potential treatment option for inflammatory lung diseases. Based on our data showing differences in the differentiation potential of bone marrow and lung MSC, we would suggest that pulmonary-derived MSC might be more appropriate for the treatment of lung diseases. It is tempting to speculate that lung-derived MSC could be used as a cell-based therapy for treatment of lung diseases like chronic rejection. Lung-derived MSC for clinical use could, for example, be generated from normal, non-diseased human adult or fetal lungs, the latter of which is an excellent source for MSC.^41^ However, the availability of both cell sources is limited and, furthermore, the use of fetal tissues raises ethical considerations. Alternative approaches such as iPS-derived MSC might be alternatives and should thus be investigated. Cultured lung-derived MSC have been proved to inhibit T-cell proliferation;^42^ however, the in vitro and in vivo immunomodulatory effects of lung MSC have to be studied in more detail in comparison with bone marrow-derived cells before any final conclusions can be drawn.

In summary, our study demonstrates that tissue-resident MSC can be isolated from central and peripheral transbronchial lung biopsies of lung-transplanted patients. Furthermore, we show—for the first time—that primary lung mesenchymal progenitor cells are highly enriched in the CD90/CD105 double positive cell fraction and that these cells are located perivascularly. Cultured lung MSC displayed multilineage potential both in vivo and in vitro*,* but showed impaired bone formation in vivo, indicating that lung-derived MSC are phenotypically and functionally different from MSC from other sources, such as bone marrow, and might therefore be a better alternative for cell-based therapy of lung diseases like obliterative bronchiolitis.

## Supplementary Material

Web supplement

Web supplement

Web supplement

Web supplement
