# Supplementary material for: Primary mesenchymal stem cells in human transplanted lungs are CD90/CD105 perivascularly located tissue-resident cells
Source: BMJ Open Respir Res. 2014 May 17;1(1):e000027. doi: 10.1136/bmjresp-2014-000027 (PMC4212711; doi:10.1136/bmjresp-2014-000027)

SUPPLEMENTARY METHODS

Patients

Biopsies were obtained from seventeen males and sixteen females, mean age at transplantation was 47.6 years (range: 20-64). The patients were transplanted because of cystic fibrosis (n=9), alpha-1-antitrypsin deficiency (n=6), chronic obstructive pulmonary disease/emphysema (n=5), lung fibrosis (n=5), pulmonary arterial hypertension (n=2), bronchiectasis (n=2), development of bronchiolitis obliterans syndrome after Graft-Versus-Host disease as a complication of a stem cell transplantation (n=2), sarcoidosis (n=1) and Eisenmenger's syndrome (n=1). Bronchoscopies were performed either as a routine evaluation (3, 6 or 12 months after transplantation) or due to suspicion of acute or chronic rejection. Twenty-nine of the patients underwent double transplantation, three a single and one patient had a heart and lung transplantation. Twenty-two of the patients were CMV seropositive and eleven were negative (Supplement Table 1). Some of the biopsies were embedded with paraffin and sectioned. The local ethics committee in Lund, Sweden approved the study and all patients gave their written informed consent to participate in the study (2005-560).

Gene expression analysis

Primers used were as follow: for *GAPDH* F 5'- CACTCCACCTTTGACGC -3', R 5'- GGTCCAGGGGTCTTACTCC -3', for *ACAN* F 5'- ACTCTGGGTTTTCGTGACTCT -3', R 5'- ACACTCAGCGAGTTGTCATGG -3', for *ALPL* F 5'- AGCTGAACAGGAACAACGTG -3', R 5'-

CAGCAAGAAGAAGCCTTTGG -3' and for *PPARG* F 5'-TGCAGGTGATCAAGAAGACG -3', R 5'- GAAGGGAAATGTTGGCAGTG -3'.

Statistics

CFU-F data were analyzed statistically by using Mann-Whitney U-test to compare between central and peripheral CFU-F. Results are presented as median and range. Analyses were performed with GraphPad Prism software version 5.0c (GraphPad Software, San Diego, CA, USA). P-value < 0.05 were considered as significant.

Two Kaplan Meier analyses with BOS as outcome variable and time (in years from 0 to 6 years), status (censored observations=1) and with the groups CFU-F central (<50 = 0 versus >50 = 1) and CFU-F peripheral (<10 = 0 versus >10=1) respectively were conducted.(1) To compare factor levels Breslow test (Generalized Wilcoxon) was used due to violation of survival test assumptions. Breslow is a test for comparing the equality of survival distributions and the time points are weighted by the number of cases at risk at each time point. The analyses were conducted with the IBM SPSS Statistics 19.0 software package. P-values ≤ 0.05 were considered as significant. Furthermore, data were analyzed for correlation by a predictive model built on multivariate logistic regression. The dependent variables were CFU-F groups (central: <50 versus >50, peripheral: <10 versus >10), and independent variables were BOS at bronchoscopy, BOS at study end, age of recipient at transplantation, and gender of the recipient. The regression analyses were conducted with the IBM SPSS Statistics 19.0 software package. P-values ≤ 0.05 were considered as significant.

SUPPLEMENTARY REFERENCES

1. Chan YH. Biostatistics 203. Survival analysis. Singapore Med J. 2004;45(6):249-56.

SUPPLEMENTARY FIGURES

Supplement figure 1. Tissue-resident MSC do not correlate with BOS development after lung transplantation. Central and peripheral transbronchial CFU-F numbers were analyzed by Kaplan Meier analysis in order to evaluate if CFU-F numbers correlated with development of BOS after lung transplantation. No correlations were seen between CFU-F numbers and time to BOS development (central CFU-F p=0.508 and peripheral transbronchial p=0.541). CFU-F, colony-forming unit-fibroblast; BOS, bronchiolitis obliterans syndrome.

A

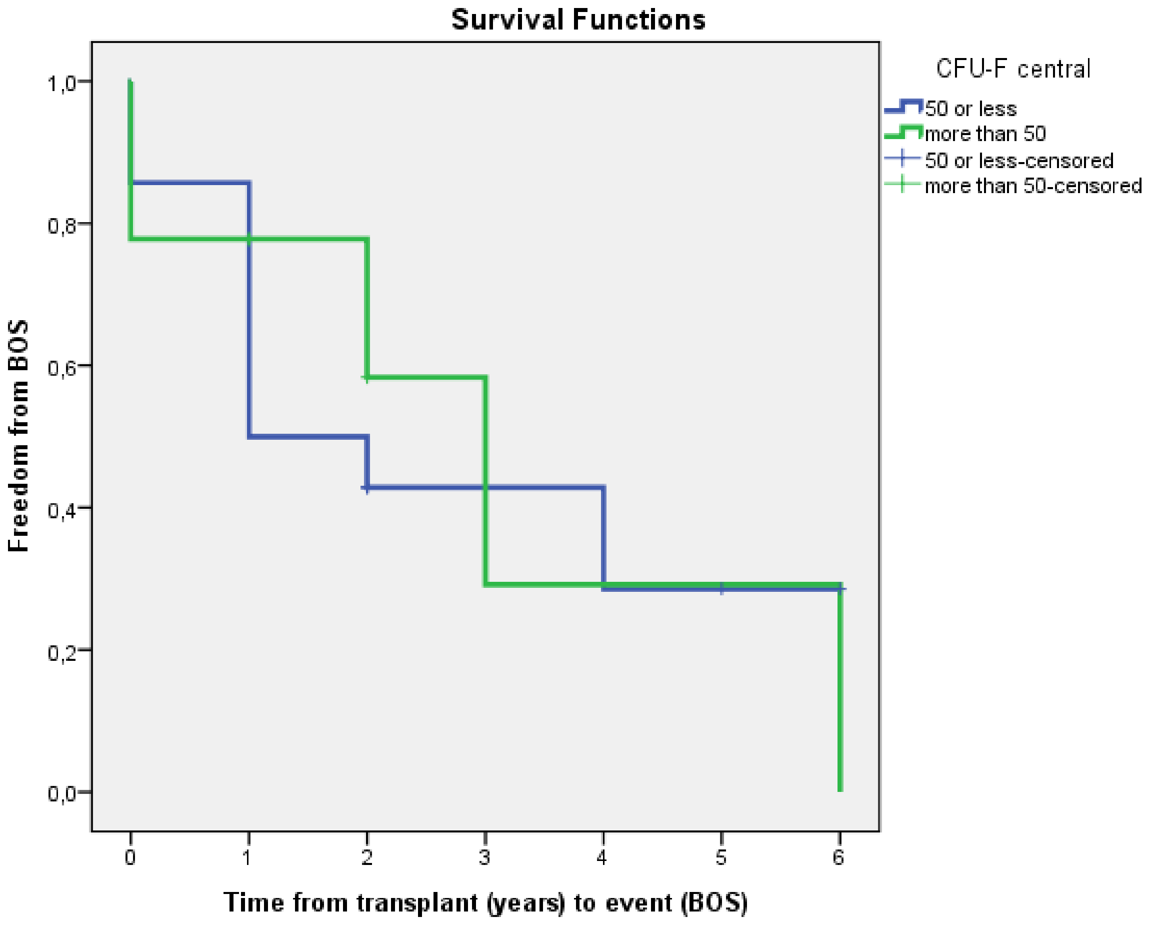

CFU-F	0	1	2	3	4	5	6
≤50, n=14	12	7	3	3	2	1	0
>50, n=9	7	4	2	1	1	1	0

Number of patients with freedom from BOS

B

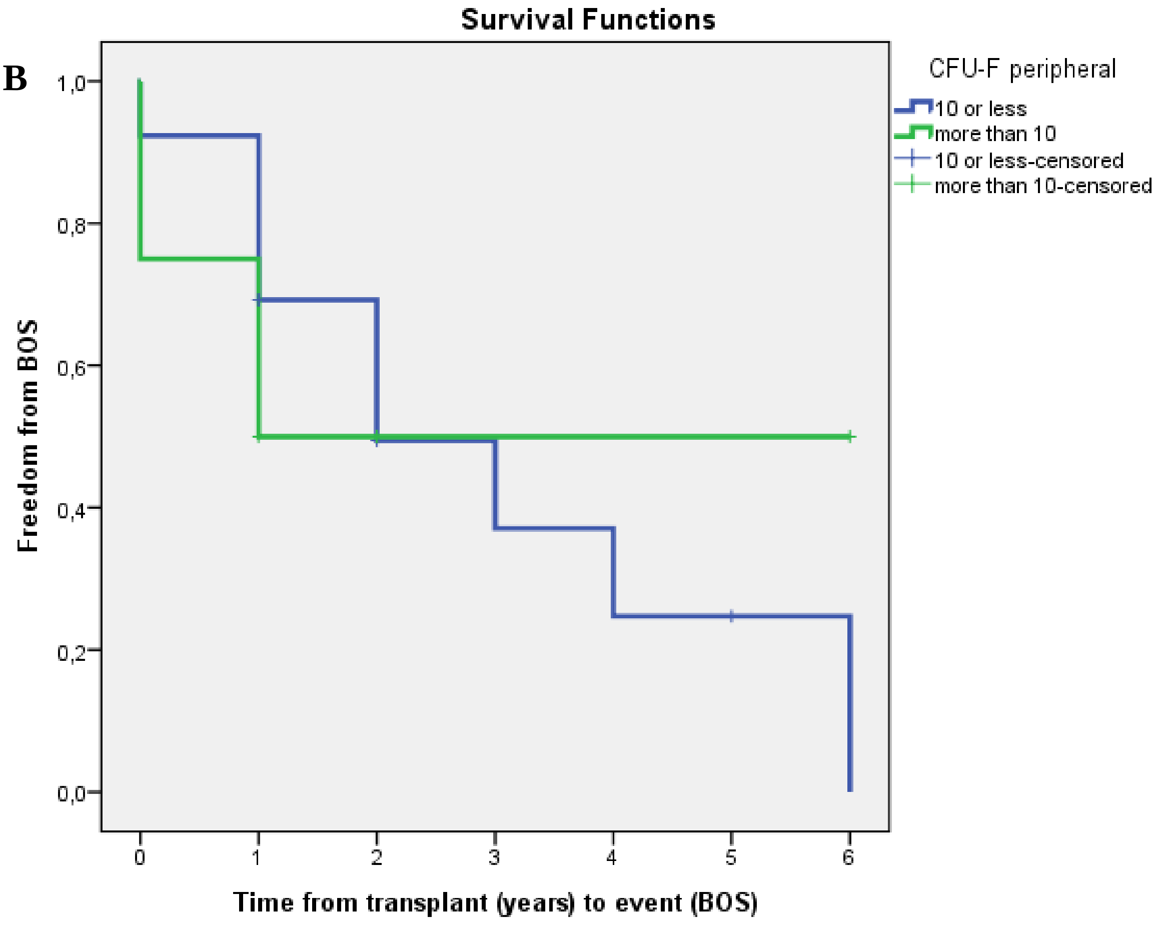

CFU-F	0	1	2	3	4	5	6
≤10, n=13	12	7	4	3	2	1	0
>10, n=8	6	3	1	1	1	1	0

Number of patients with freedom from BOS

Supplement figure 2. Cultured peripheral transbronchial lung-derived MSC show a typical surface marker profile. Cultured MSC (passage 3 and 4) isolated from peripheral transbronchial biopsies of four lung- transplanted patients were harvested and stained with antibodies and analyzed with flow cytometry. A representative peripheral lung-MSC surface marker profile from one of the patients is presented. Sample: red shaded area. Isotype control: grey line.

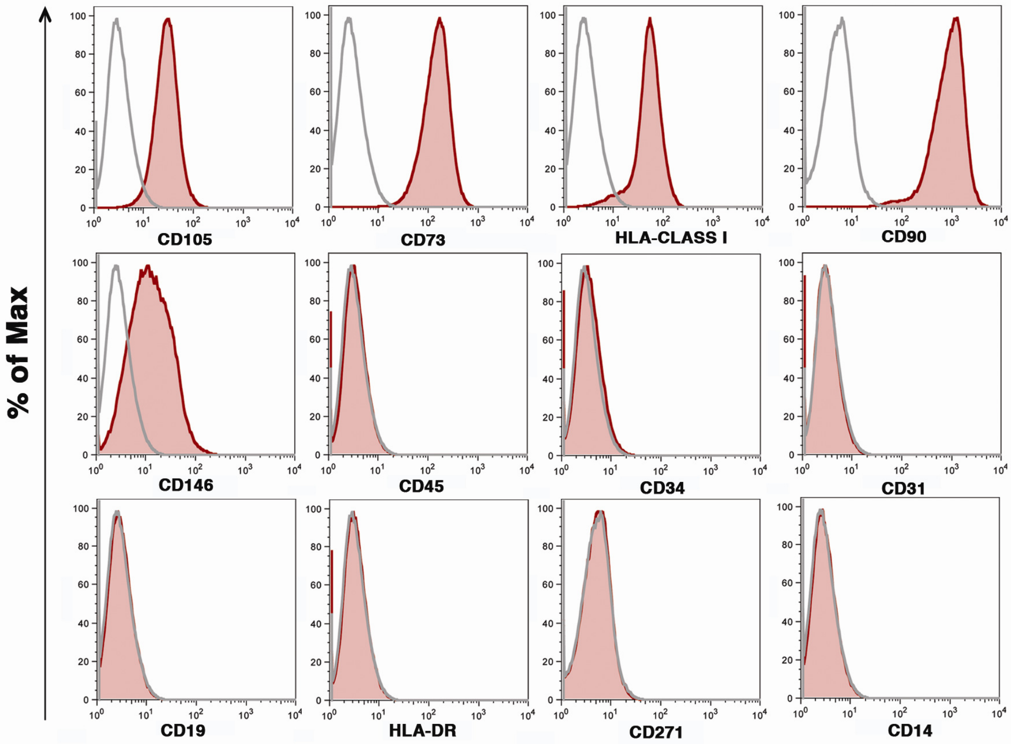

% of Max
CD105
CD73
HLA-CLASS I
CD90
CD146
CD45
CD34
CD31
CD19
HLA-DR
CD271
CD14

Supplement figure 3. Sorting of lung cells based an CD271 and CD146 expression does not allow to enrich for colony forming cells. Lung cells were isolated from central and peripheral transbronchial lung biopsies of lung- transplanted patients (n=4) and stained with directly conjugated antibodies against CD271- APC and CD146-PE. Cells were sorted on a FACSAria I or FACSAria III cell sorter. Single stained cells were used to set up sorting gates and doublets were excluded by gating on SSC- H versus SSC-W (B) and FSH-H versus FSC-W (C). The CD271+/CD146+, CD271- /CD146+, CD271+/CD146+ and CD271-/CD146- were collected and assayed for CFU-F content (D). After 14 days of culture plates were stained with Crystal violet and colonies were counted (E). CFU-F = Colony-forming unit-fibroblast. Colonies with ≥ 20 cells were counted as CFU-F.

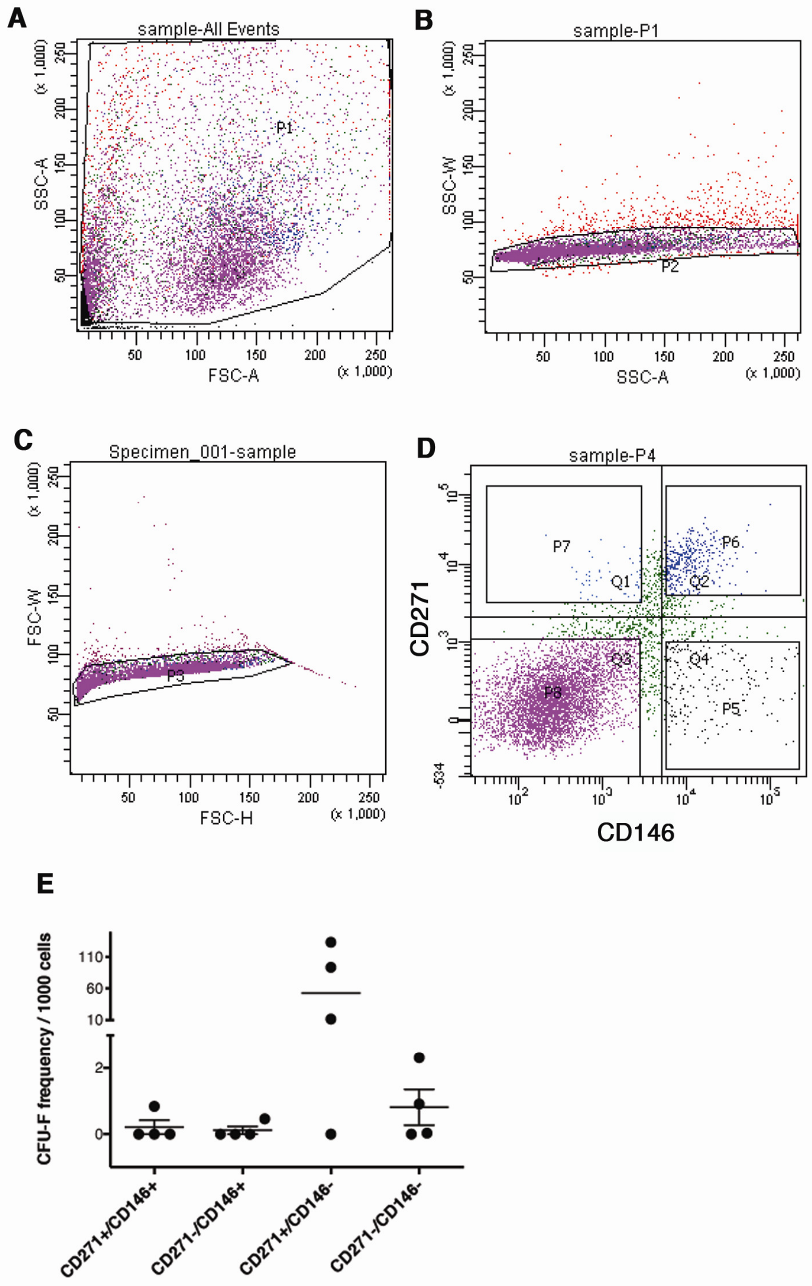
A
sample-All Events
P1
SSC-A
FSC-A
(x 1,000)
B
sample-P1
P2
SSC-W
SSC-A
(x 1,000)
C
Specimen_001-sample
P3
FSC-W
FSC-H
(x 1,000)
D
sample-P4
P7
P6
Q1
Q2
Q3
Q4
P8
P5
CD271
CD146
E
CFU-F frequency / 1000 cells
CD271+/CD146+
CD271-/CD146+
CD271+/CD146-
CD271-/CD146-

Supplement figure 4. Sorting strategy for primary CD90+/CD105+ MSC. Single cell suspensions were isolated from central and peripheral transbronchial lung biopsies of lung- transplanted patients, stained with directly conjugated antibodies against CD90- APC (Thy-1) and CD105-FITC (Endoglin), and sorted by fluorescent activated cell sorting. Lung cells were first gated based on forward/side scatter properties (A). Single color stained cells were used to set up sorting gates and doublets were excluded by gating on SSC-H versus SSC-W (B) and FSH-H versus FSC-W (C). Staining with 7-amino-actinomycin D (7-AAD) was used for dead cell exclusion (D). CD90+/CD105+, CD90-/CD105+, CD90+/CD105- and CD90-/CD105- cell populations were sorted and assayed for CFU-F content (E).

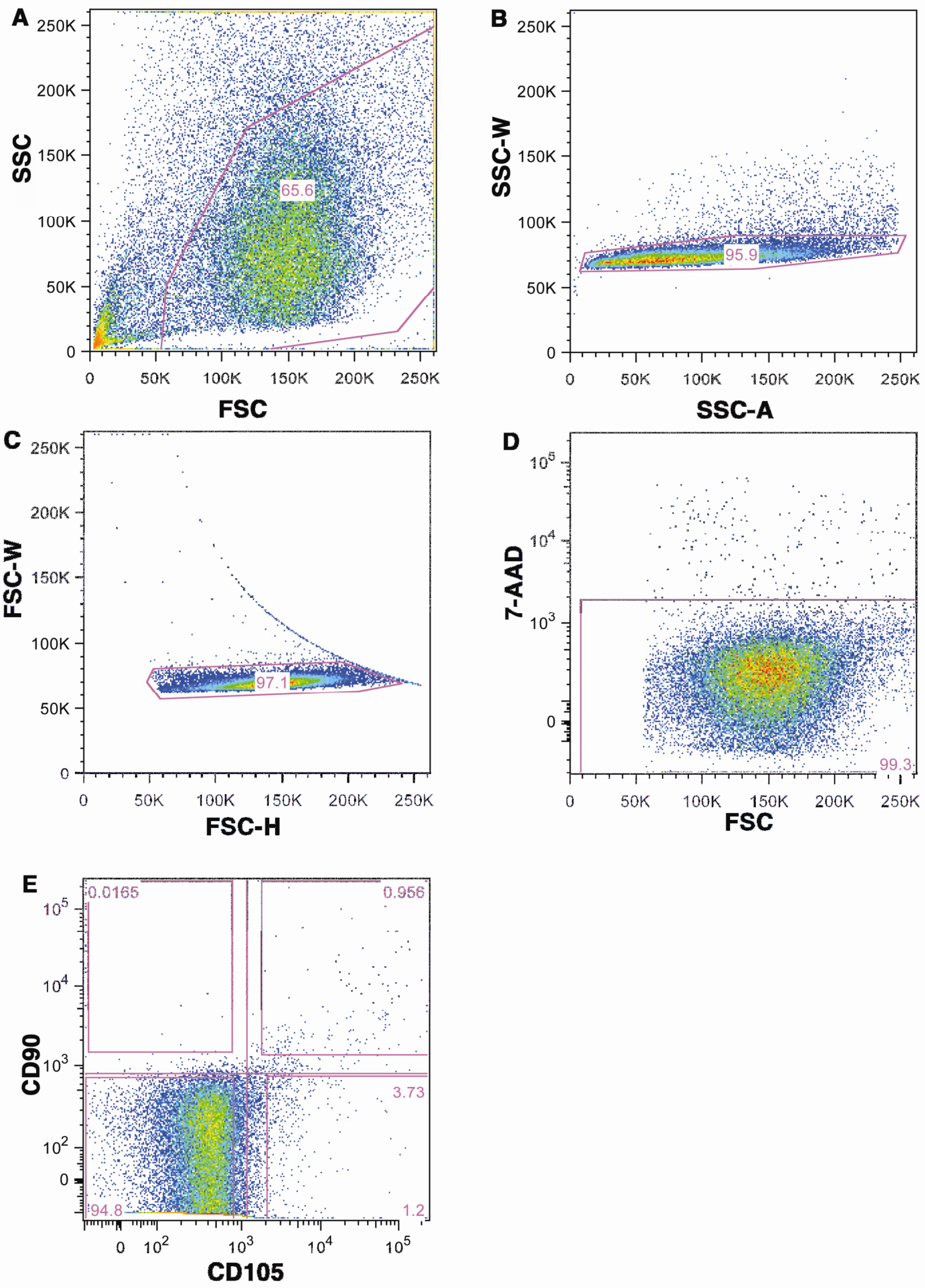
A
SSC
FSC
65.6
B
SSC-W
SSC-A
95.9
C
FSC-W
FSC-H
97.1
D
7-AAD
FSC
99.3
E
CD90
CD105
0.0165
0.956
3.73
94.8
1.2

Supplement figure 5. Isotype controls for immunofluorescent staining.

Paraffin embedded lung biopsies obtained from lung-transplanted patients were sectioned and stained with isotype controls. (A) Merged picture of DAPI staining (B) and a mouse IgG1 isotype control (C). (D) Merged picture of DAPI (E) and rabbit IgG isotype control staining (F).

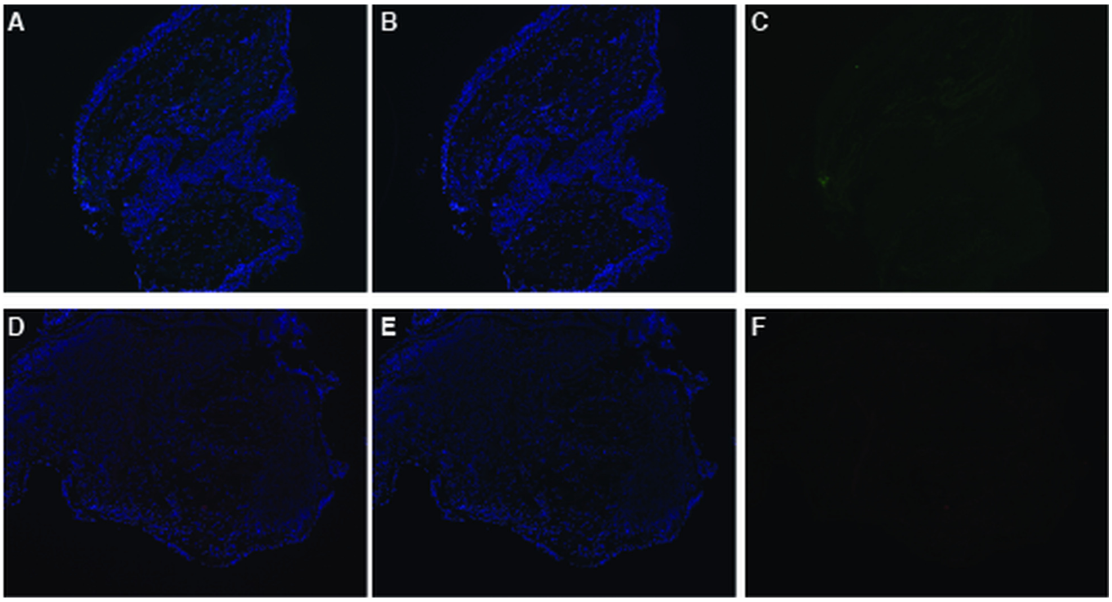

Supplement: Web supplement [file bmjresp-2014-000027-s1.pdf]
