# Supplementary material for: Primary mesenchymal stem cells in human transplanted lungs are CD90/CD105 perivascularly located tissue-resident cells
Source: BMJ Open Respir Res. 2014 May 17;1(1):e000027. doi: 10.1136/bmjresp-2014-000027 (PMC4212711; doi:10.1136/bmjresp-2014-000027)
Supplement: Web supplement [file bmjresp-2014-000027-s2.pdf]

SUPPLEMENT TABLE 1. CHARACTERISTICS OF LUNG TRANSPLANTED PATIENTS

Underlying disease	Age at LTP (yrs.)	Gender (F/M)	Type of LTP (S/D/HL)	CMV (p/n)	FEV1 (L) at BRO	BOS grade at BRO	BOS grade at study end (mo. after LTP)	Assays C/S
CF	49	M	D	p	2.7	0	1 (36)	C
CF	40	M	D	p	4.4	0	1 (65)	C
CF	20	F	D	n	1.6, 1.5	0, 0	1 (35)	C,C
CF	31	M	D	n	4.0, 4.55*	n/a	n/a	C
CF	45	F	D	n	2.2 *	n/a	n/a	C, S
CF	48	M	D	p	2.8, 3.1	0, 0	0 (21)	C, S
CF	46	M	D	n	n/a	n/a	n/a	C, S
CF	42	M	D	n	2.8	0	†	S
CF	41	F	D	n	2.2*	n/a	n/a	S
A1AT	49	M	D	n	3.3	0	0 (160)	C
A1AT	47	F	D	n	2.2, 1.8, 2.1	0, 1, 1	2 (27)	C,C,C&S
A1AT	56	F	D	p	2.7	0	0 (26)	C
A1AT	54	F	D	p	1.3	1	1 (29)	C
A1AT	56	M	D	n	1.9	3	3 (49)	S
A1AT	41	F	D	p	4.7	0	0 (7)	S
COPD/Emph	64	M	D	n	1.7	0	1 (37)	C
COPD/Emph	55	F	D	p	2.2, 2.2	0, 0	0 (30)	C,C
COPD/Emph	63	F	D	p	2.6, 2.6	0, 1	†	C,C&S
COPD/Emph	58	M	D	p	2.7, 2.7, 2.6	0,0,0	Re-LTP	C,C,C,S
COPD/Emph	61	F	S	p	0.62	3	3 (37)	C
Lung fibrosis	61	M	D	p	3.0	0	0 (18)	C
Lung fibrosis	57	F	S	p	1.2	2	†	C
Lung fibrosis	58	M	D	p	2.2	0	1 (22)	C &S
Lung fibrosis	62	M	D	n	2.8	0	0 (16)	S
Lung fibrosis	53	M	D	p	2.5	1	1 (35)	S
PAH	26	F	D	p	0.7	3	3 (221)	C
PAH	35	F	D	p	1.5, 1.63	1, 1	1 (27)	C&S,S
Bronchiectasis	34	M	D	p	1.9, 2.3	0, 0	2 (29)	C,C
Bronchiectasis	60	M	D	p	2.1	1	2 (33)	C
BOS GvH Stem cell TP	30	M	D	p	3.3	1	2 (195)	C
BOS GvH Stem cell TP	33	F	D	p	2.4	0	3 (40)	C
Sarcoidosis	57	F	S	p	1.0	2	2 (86)	C
Eisenmenger´s syndrome	40	F	HL	p	1.7	0	1 (26)	C

CF, cystic fibrosis; A1AT, alpha-1-antitrypsin deficiency; COPD/Emph, chronic obstructive pulmonary disease/emphysema;

PAH, pulmonary arterial hypertension; GvH, Graft versus host disease; LTP, lung transplantation; F, female; M, male; S, single transplantation;

D, double transplantation; HL, heart- and lung transplantation; CMV,cytomegalovirus status; p positive (IgG towards CMV) n negative (lack of IgG towards CMV) FEV_1, forced expiratory volume in 1 second; L, liter;; BOS, bronchiolitis obliterans syndrome; BRO, bronchoscopy; yrs., years; mo., month; TP, transplantation; n/a, not applicable; *, only one or two FEV1 value available; †, deceased; Re-LTP, re-lung transplantation; C, cells were used for colony-forming unit fibroblast assay;

S, cells were used for fluorescence activated cell sorting
