# Supplementary material for: Primary mesenchymal stem cells in human transplanted lungs are CD90/CD105 perivascularly located tissue-resident cells
Source: BMJ Open Respir Res. 2014 May 17;1(1):e000027. doi: 10.1136/bmjresp-2014-000027 (PMC4212711; doi:10.1136/bmjresp-2014-000027)
Supplement: Web supplement [file bmjresp-2014-000027-s3.pdf]

SUPPLEMENT TABLE 2. LOGISTIC REGRESSION (ENTER MODEL) ANALYS (n=24)

	**Central CFU-F as dependent variable (0=50 or below; 1= over 50 colonies), n=29**		**Peripheral CFU-F as dependent variable (0=10 or below; 1= over 10 colonies), n=30**	
	Odds ratio (95% CI)	P-value	Odds ratio (95% CI)	P-value
**BOS bronchoscopy**	8.26 (0.60-112.85)	0.114	1.75 (0.10-30.0)	0.697
**BOS at study end**	0.20 (0.02-2.52)	0.213	0.19 (0.01-3.09)	0.245
**Age recipient at LTP**	0.99 (0.93-1.06)	0.802	0.91 (0.82-1.10)	0.085
**Gender**	1.35 (0.18-9.98)	0.770	5.44 (0.51-58.41)	0.162

Central CFU-F: Hosmer & Lemeshow Goodness of Fit Test chi-square 4.99, p-value 0.758 (poor fit is indicated by a p-value below 0.05). Nagelkerke R Square = 17.8%.
Peripheral CFU-F: Hosmer & Lemeshow Goodness of Fit Test chi-square 9.97, p-value 0.267. Nagelkerke R Square = 31.8%. CI = Confidence interval, BOS= bronchiolitis obliterans syndrome, LTP = lung transplantation, p-value ≤ 0.05 were considered significant.
