# Supplementary material for: Primary mesenchymal stem cells in human transplanted lungs are CD90/CD105 perivascularly located tissue-resident cells
Source: BMJ Open Respir Res. 2014 May 17;1(1):e000027. doi: 10.1136/bmjresp-2014-000027 (PMC4212711; doi:10.1136/bmjresp-2014-000027)
Supplement: Web supplement [file bmjresp-2014-000027-s4.pdf]

SUPPLEMENT TABLE 3. FISH ANALYSIS OF CULTURED LUNG-DERIVED

Subject number	Location of biopsy (C/P)	Gender subject	Gender donor	Gender lung-MSC	Time after transplantation	BOS grade at transplantation
1	P	XY	XX	99 % XX	5.9 mo.	BOS 0
1	C	XY	XX	100 % XX	5.9 mo.	BOS 0
2	P	XY	XX	97 % XX	6.0 mo.	BOS 0
2	C	XY	XX	93 % XX	6.0 mo.	BOS 0
3	C	XY	XX	98 % XX	3 yrs. 3.8 mo.	BOS 0
4	P	XX	XY	98 % XY	15 yrs. 11.9 mo.	BOS 3
4	C	XX	XY	95 % XY	15 yrs. 11.9 mo.	BOS 3
5	P	XX	XY	97 % XY	3.0 mo.	BOS 1
5	C	XX	XY	95 % XY	3.0 mo.	BOS 1
6	C	XX	XY	96 % XY	3.3 mo.	BOS 0
7	P	XX	XY	93 % XY	5.1 mo.	BOS 0
7	C	XX	XY	98 % XY	5.1 mo.	BOS 0

FISH, fluorescence in situ hybridization; C, central; P, peripheral transbronchial; XY, male; XX, female; MSC, mesenchymal stromal cell; mo., months; yrs., years; BOS, bronchiolitis obliterans syndrome
